# Effects of Supercritical CO_2_ on the Pore Structure Complexity of High-Rank Coal with Water Participation and the Implications for CO_2_ ECBM

**DOI:** 10.1021/acsomega.3c01486

**Published:** 2023-05-19

**Authors:** Xicong Ma, Yi Du, Changqing Fu, Huihuang Fang, Haonan Wei, Zhejun Pan, Shuxun Sang, Junying Zhang

**Affiliations:** † National and Local Joint Engineering Research Center for Carbon Capture Utilization and Sequestration & State Key Laboratory of Continental Dynamics, Department of Geology, Northwest University, Xi’an 710069, China; ‡ College of Geology and Environment, Xi’an University of Science & Technology, Xi’an 710054, China; § State Key Laboratory of Coal Combustion, School of Energy and Power Engineering, Huazhong University of Science & Technology, Wuhan 430074, China; ∥ School of Earth and Environment, Anhui University of Science and Technology, Huainan, Anhui 232001, China; ⊥ Key Laboratory of Continental Shale Hydrocarbon Accumulation and Efficient Development, Ministry of Education, Northeast Petroleum University, Daqing 163318, China; # Jiangsu Key Laboratory of Coal-based Greenhouse Gas Control and Utilization, China University of Mining and Technology, Xuzhou, Jiangsu 221008, China

## Abstract

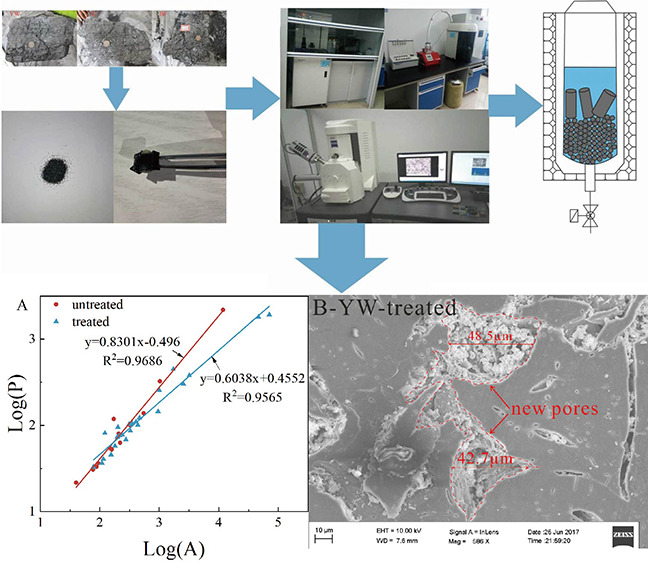

To reveal how mineral changes affect a coal pore structure in the presence of water, an autoclave was used to carry out the supercritical CO_2_ (ScCO_2_)-H_2_O-coal interaction process. To reveal the changes in pore complexity, mercury intrusion capillary pressure (MICP), low-pressure nitrogen adsorption, CO_2_ adsorption, and field emission scanning electron microscopy (FESEM) experiments were combined with fractal theory. The experimental data of MICP show that the MICP data are meaningful only for the pore fractal dimension with pore sizes >150 nm. Therefore, the pores were classified into the classes >150, 2–150, and <2 nm. The results show that the pore volume and specific surface area of the coal increased significantly after the reaction. ScCO_2_-H_2_O can cause the formation of many new pores and fractures in the coal. The presence of H_2_O may increase the potential for the injection of CO_2_ into the coal seam. The complete dissolution of calcite surfaces caused a significant increase in the pore volume and specific surface area of the pores >150 nm. The morphologies of these pores are controlled by the morphologies of the complete dissolution carbonate particles. The pore morphologies were relatively uniform, and the fractal dimensions decreased. However, the incomplete dissolution of calcite leads to irregular variations in the morphologies for the pores in the 2–150 nm pore size range. The pore morphologies that are produced by incompletely dissolved calcite particles are more complex, which increases the fractal dimensions after the reaction. The fractal dimensions of the pores <2 nm decreased after the reaction, indicating that the newly generated micropores were more uniform and had regular pore morphologies.

## 1 Introduction

The consumption of various resources has gradually increased with industrial and technological development. This development has led to the production and release of a large amount of CO_2_. Since CO_2_ is the main greenhouse agent, the reduction of CO_2_ has become a grave concern.^1^ Current on-site CO_2_ storage options for carbon capture include oil and gas reservoirs, deep salt formations, and abandoned or unmineable coal mines.^2,3^ The CO_2_ molecules will enter the pores in the coal and will be adsorbed after the CO_2_ is injected into the coal seam. CO_2_ competes with methane for adsorption sites and will replace the methane in the coal pores since the adsorption capacity for CO_2_ is higher than for CH_4_.^4^ The coal seams that have been used for CO_2_ geological storage up to now have all been larger than 800 m, with temperatures and pressures that are higher than the supercritical state of CO_2._^5^ The CO_2_ that is being injected is in a supercritical state and can weaken the adsorption of CH_4_. This increases the desorption of CH_4_ and increases the production of coalbed methane.^6^ The storage of CO_2_ in coal seams is, therefore, very promising and can aid in additional energy development from coalbed methane reservoirs.^7−11^

Coalbed methane is mainly extracted by water injection, where the water injection capacity is determined by the permeability of the coal formation. Many studies that have been conducted on the permeability of coal reservoirs show that the coal formation permeability is critical to the injection and seepage capacity of coal reservoirs.^11−13^ The pore structure of the geological formations determined the degree of gas occurrence and seepage capacity of the formations.^14−17^ Therefore, the coal pore structure after CO_2_ injection is the main focus of CO_2_ storage in coal seams. Many factors influence the coal pores after CO_2_ injection. This includes coal maturity, reaction temperature, and pressure conditions. When the CO_2_ reacts with the minerals in the coal, it dissolves some of the minerals and produces new minerals. These changes affect the pore structure of the coal.^18,19^ ScCO_2_ has different effects on pores of the different ranks of coal, which are related to the different pore sizes. CO_2_ injection mainly affects the macropores in low-ranking coals, while for high-ranking coals, it is mainly micropores that determine the pore volume and specific surface area.^20,21^

Fractal dimensions can be used to characterize the roughness and complexity of the pore structure. More complex pore structures with irregular pore surfaces have a larger fractal dimension.^22^ The fractal dimensions of coal are to a large degree affected by the injection of ScCO_2_ into the coal formation.^23,24^ Pore roughness is also an important factor affecting gas adsorption. The fractal dimension of coal pores has therefore become an important research focus of CO_2_ sequestration.^25^ Some of the ways to obtain the fractal dimensions of coal include mercury intrusion capillary pressure (MICP), low-pressure nitrogen/carbon dioxide adsorption, scanning electron microscopy (SEM), and small-angle X-ray scattering (SAXS).^26−29^ Current research on the changes in the fractal dimensions of coal after CO_2_ injection shows that the fractal dimension of the pores will increase, and the surface pores will be rougher after the reaction. These factors combine to make the coal layers more conducive to CO_2_ sequestration.^30,31^ The fractal dimension is also affected by the burial depth and maturation degree of the coal. The deeper the coal is buried, the larger the temperature increase and the less the coal fractal dimension.^32^ The fractal dimensions are calculated through MICP and N_2_ and CO_2_ adsorption. High-precision and high-resolution SEM images can be used to characterize the structural information of the pores.^24^ There is a logarithmic relationship between the fractal dimension and porosity of coal pores by using the image fractal results and porosity.^33^ The research of Li and Wu shows that the pore characteristics obtained by the fractal information from the SEM images do not differ much from those obtained by traditional methods.^34^ This shows that fractal images are useful for studying pore changes.

In previous studies, the pore structure and fractal dimensions were investigated via MICP, LP-N_2_ adsorption, and CO_2_ adsorption. SEM was used in some of the studies to investigate the link between mineral variations and the pore structure. However, only a few studies used MICP, gas adsorption, and SEM in combination for pore structure analysis. Three high-ranking coals with different maturities were selected for this study, and MICP, gas adsorption testing, and SEM experiments were used to study the pore structure, fractal dimensions, and mineral changes before and after ScCO_2_ treatment. This information provides theoretical guidance for the geological storage of CO_2_.

## 2 Experiments and Methods

### 2.1 Sample

**Figure 1:**
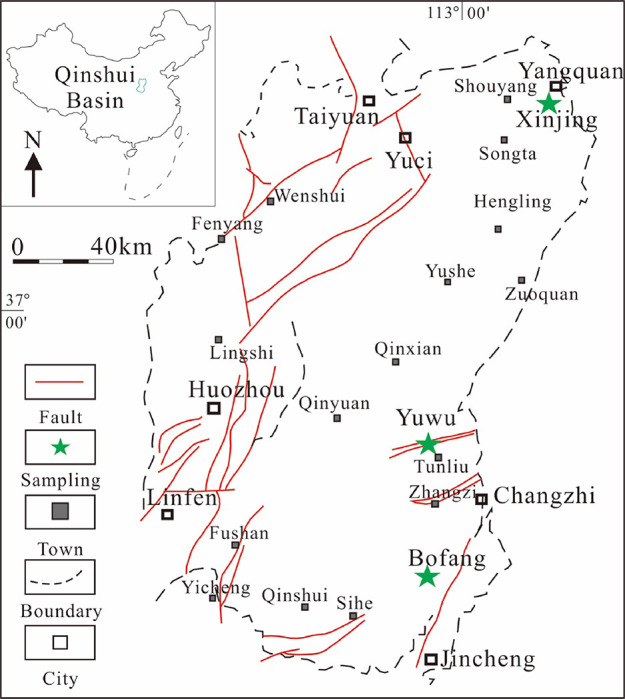
Location of the Qinshui Basin and sampling sites.^35^

**Figure 2:**
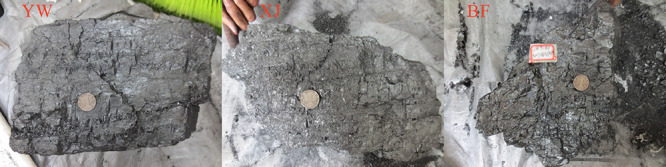
Original sample.

The Qinshui Basin is the first CO_2_-ECBM test site in China, where the high-ranking coal is more developed than in other areas. The high-ranking coal from the Qinshui Basin was therefore selected for this study. All the samples used in this study were collected from the Qinshui Basin in the southeast of Shanxi Province. The study area is between 35°–38° N, and 112°00′–113°50′ E. The samples were collected from the Yuwu mine (YW), Xinjing mine (XJ), and Bofang (BF) mine (Figure 1). The YW sample was primary structural coal with a high degree of hardness and a low degree of fracture development. The XJ samples were fragmented and had homogeneous band-like structures and massive-horizontal bedding structures. The XJ samples had relatively well-developed endogenous fissures. The BF sample was disintegrated coal, with a homogeneous structure and well-developed fractures (Figure 2). After the samples were collected, they were wrapped in plastic and placed in a sealed bag to prevent oxidation and other effects from changing the properties of the samples. All the experimental coal samples were gray-black anthracite coal with a high degree of metamorphism.

Table 1 shows the depth and basic information of the samples. A Leitz Orthoplan microscope equipped with a photomultiplier tube was used to determine the maximum vitrinite reflectance of the sample. The maximum vitrinite reflectances (*R*_o,max_) of the three samples were 2.19, 2.64, and 2.83%, respectively. All these samples can be classified as high-ranking metamorphic coals.

**Table 1:** Maximum Vitrinite Reflectance, Sampling Depths, Total Sulfur, and Ultimate and Proximate Analyses of the Tested samples^35^^a^

	*R*_o,max_	sampling depth	proximate analyses (%)			ultimate analyses (%)				
sample	(%)	(m)	*M*_ad_	*A*_ad_	*V*_daf_	*S*_t,d_	*O*_daf_	*C*_ad_	*H*_ad_	*N*_ad_
YW	2.19	539	1.10	11.98	13.44	0.28	2.98	93.45	2.15	1
XJ	2.64	585	1.66	10.02	10.10	0.33	3.05	91.52	3.96	1.06
BF	2.83	273	2.05	9.40	9.86	0.32	2.42	91.82	3.85	1.06

a *R*_o,max_ is the mean maximum reflectance values of vitrinite, *M*_ad_ is the moisture, *A*_ad_ is the ash yield, *V*_daf_ is the volatile matter, *S*_t,d_ is the total sulfur content, *O*_daf_ is the oxygen content, *C*_ad_ is the carbon content, *H*_ad_ is the hydrogen content, *N*_ad_ is the nitrogen content, “ad” is the air-drying base; “daf” is the dry ash-free basis.

The samples were subjected to approximate based on the ASTM standards, and the ash yield, moisture content, and volatile organic matter were analyzed by using a Vario macro elemental analyzer. Table 2 shows the detailed results.

**Table 2:** Organic Matter Composition and Mineral Composition of the Test Samples^36^

	organic matter (vol %)		relative content of mineral components (%)			
sample	vitrinite	inert group	total	clay	carbonate	other
YW	75.56	24.44	7.7	58.43	9.67	31.9
XJ	70.70	29.30	11.78	75.14	4.07	20.79
BF	71.72	28.28	9.63	73.6	4.12	22.28

A QuantaTM 250 scanning electron microscope from FEI, USA was used to analyze the samples before and after the reaction. The small mineral particles in the coal complicated efforts to accurately locate the minerals. All the photos that were taken were therefore first taken at low magnification in the backscattering mode during the FESEM analyses. Thereafter, the mineral composition was preliminarily identified by the Advanced Mineral Identification and Characterization System (AMICS). To further observe the characteristic minerals in secondary electron mode, the position of the characteristic minerals in the photo was then marked and gradually enlarged. The surface of the sample must be sprayed with gold to conduct electricity when performing FESEM analyses. However, when the coal surface has been sprayed with gold, it will be covered, thereby hindering the reaction of the sample with ScCO_2_. Gold spraying treatment can therefore not be done before the reaction. Conductive glue must be pasted on the edge of the sample to export the charge and to ensure accurate observation results.

**Table 3:** Changes in Pore Connectivity

	effectively connection of pores (mL/g)		percentage of effectively connected pores		noneffectively connected pores (mL/g)		percentage of noneffectively connected pores	
sample	untreated	treated	untreated	treated	untreated	treated	untreated	treated
YW	0.0189	0.02094	54.47%	44.27%	0.0158	0.02636	45.53%	55.73%
XJ	0.0193	0.0204	55.78%	65.59%	0.0153	0.0107	44.22%	34.41%
BF	0.0195	0.02296	56.52%	42.60%	0.015	0.03094	43.48%	57.40%

**Table 4:** Fractal Dimensions of MICP, where *D*_1_ Represents >150 nm Pore Fractal Dimension and *D*_2_ Represents <150 nm Pore Fractal Dimension

sample	*A*_1_	*D*_1_	*R*^2^	*A*_2_	*D*_2_	*R*^2^
YW-untreated	–1.0033	2.9967	0.9275	–0.3107	3.6893	0.7248
YW-treated	–1.3378	2.6622	0.9112	–0.0619	3.9381	0.0402
XJ-untreated	–1.1666	2.8334	0.9287	–0.061	3.939	0.0859
XJ-treated	–1.0001	2.9999	0.9367	–0.2105	3.7895	0.0859
BF-untreated	–1.1409	2.8591	0.9278	–0.0393	3.9607	0.0377
BF-treated	–1.3173	2.6827	0.911	–0.1987	3.8013	0.2688

The AutoPore IV 9500 automatic experimental instrument produced by Micrometrics in the United States was used to perform the mercury intrusion capillary pressure experiment. The mercury intrusion capillary pressure experiment was tested according to the ISO15901-1:2005 international standard. The TriStar II 3020 rapid specific surface area analyzer produced by Micrometrics in the United States, was tested according to ISO 15901-2:2006 international standard, was used to perform the low-pressure nitrogen adsorption experiment. The Quantachrome Instruments 4200e physical adsorption instrument was used to carry out the CO_2_ adsorption experiments, and the adsorption experiments were carried out according to the ISO 15901-3:2007 international standard.

### 2.2 Experimental Procedures

The common distribution depth of CBM in the Qinshui Basin is 1500 m.^37,38^ Therefore, this depth was chosen for the simulation experiment. According to the ground temperature gradient in the Qinshui basin, the temperature and pressure at this depth are 50 °C and 15 MPa, respectively. CO_2_ is in a critical state at these temperature and pressure conditions. In previous studies, we measured the elemental content of the experimental water daily. Our results indicated significant fluctuations during the initial two days, followed by stabilization. Consequently, we chose a 10 day reaction time for a complete mineral reaction.^39^

**Figure 3:**
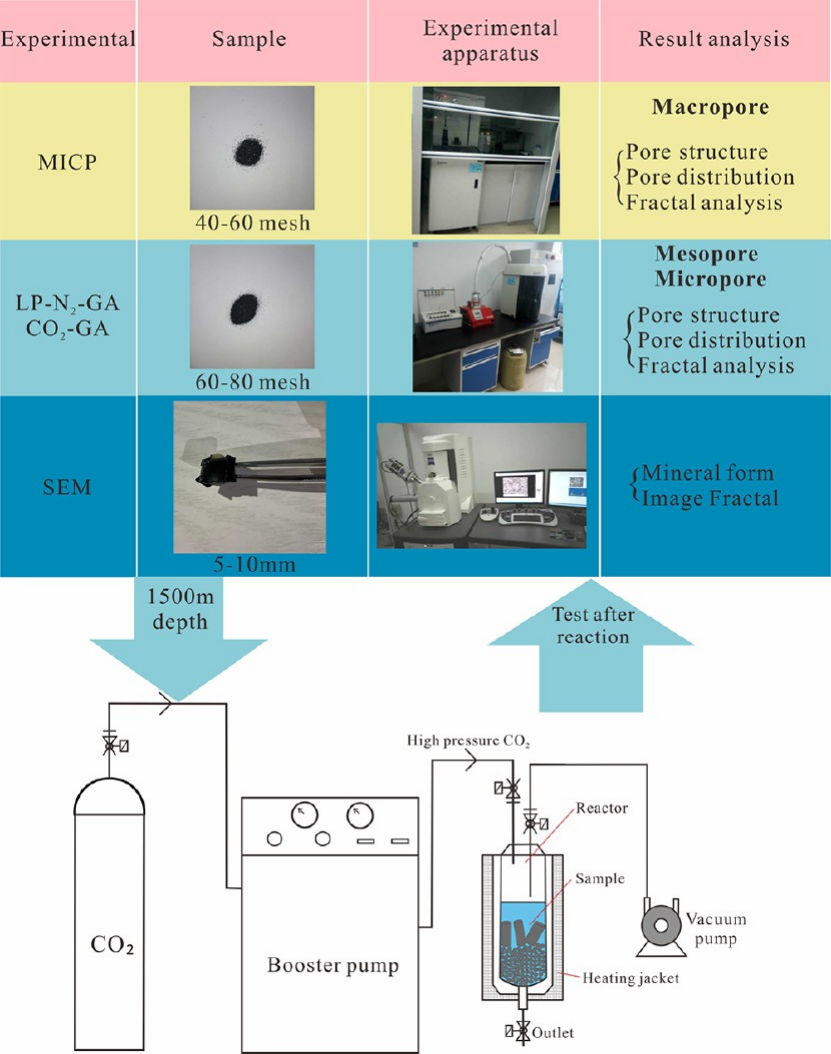
Experimental samples, equipment, and procedures.

The experimental equipment consisted of a sample chamber, pressurization system, heating system, vacuum system, fluid sample acquisition system, and control and monitoring system. An 8–18 mesh particle size for MICP analyses and a 40–60 mesh particle size for LP-N_2_ and CO_2_ adsorption were used to process the samples (Figure 3). To increase the reaction speed, 600 mL of deionized water was added to each group of experiments, which increased the solid–liquid ratio.

The experimental procedure is as follows:

(1) Clean the reactor to avoid affecting the experimental results.
(2) Put the sample into the reactor and seal the reactor.
(3) Inject CO2 until the pressure in the reactor reaches 15 MPa.
(4) Set the temperature to 50 °C and start the experiment.

### 2.3 Analytical Method

#### 2.3.1 MICP

The MICP can analyze pores ranging in size from 5.5 to 178 μm. When the mercury has a contact angle greater than 90°, it cannot enter the microcracks under zero pressure conditions. The main force provided by the surface tension of the mercury can be overcome by an external force, which creates a functional relationship between pressure and pore size. This information can be calculated using the Washburn equation, see eq 1:^40^
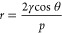
1

Here, *r* is the pore radius (μm), θ is the mercury-coal contact angle (130°), γ is the surface tension of mercury (0.48 N/m), and *p* is the mercury injection pressure.

Assuming that the pores are cylindrical, the specific surface area of the pores can be calculated by eq 2:
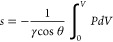
2

The fractal dimension can be calculated according to the method of Friesen and Mikula, see eq 3:^41^
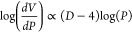
3

In eqs 2 and 3, *P* is the mercury injection pressure, and *D* is the fractal dimension.

#### 2.3.2 Gas Adsorption

Gas adsorption tests can be used to test a specific surface area and the pore distribution of gas on a solid surface at a constant temperature. A specific pressure corresponds to a specific amount of adsorption when the adsorption is at equilibrium. Changing the pressure can change the amount of gas adsorption. Pores in the range of 0.85–150 nm can be analyzed by nitrogen adsorption. Most of the problems encountered by other adsorbents, such as a lower adsorption temperature or a large molecular size that hinders its access to smaller pores, can be overcome by CO_2_.^42,43^ CO_2_ adsorption can therefore be used to study the pore structure and pore distribution of the small pores. The Brunauer–Emmett–Teller (BET) theory was used to calculate the pore surface area. However, the pore distribution was calculated using the Barrett–Joyner–Halenda (BJH) theory.^44,45^ The Frenkel–Halsry–Hill (FHH) model was used to calculate the fractal dimensions:^46−48^
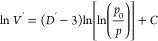
4

In eq 4, *V*^’^ is the adsorbed volume at the equilibrium pressure *p* in m^3^, *p*_0_ is the saturation pressure in MPa, *p* is the equilibrium pressure in MPa, and *C* is a constant. Two calculation methods, namely, *D* = *K* + 3 and *D* = 3*K* + 3, can be used to calculate the fractal dimension from the slope *K* of ln*V* and ln[ ln (*p*_0_/*p*)]. There is no consensus about which of the two calculation methods is better. The fractal dimension of the solid surface is 2–3, where the lower limit of 2 represents a completely smooth surface and the upper limit of 3 represents the maximum complexity allowed for the surface.^46^ For this analysis, the calculation method was guided by the obtained results.

There is a correlation between the pore volume and specific surface area of the solid porous media in the *V*-*S* model, which was first proposed by Mandelbrot and Wheeler.^49^ Several studies used the *V*-*S* model to analyze the fractal dimensions of the micropores in coal. This model is also used for fractal research in shale. Here, the *V*-*S* model is suitable for determining the fractal characteristics of the micropores, but it is less suitable for determining the fractal dimensions of the mesopores and macropores.^50^ Both coal and shale are porous sedimentary media, and the *V*-*S* model was used to determine the fractal dimensions of CO_2_ adsorption. Our study calculations showed that this model is also suitable for the fractal analysis of the coal micropores. This was calculated according to eq 5:
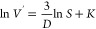
5

In eq 5, *K* is a constant, *V*^’^ is the cumulative pore volume (cm^3^/g), *S* is the cumulative specific surface area (m^2^/g), *D* is the micropore fractal dimension.

#### 2.3.3 Image Fractal

**Figure 4:**
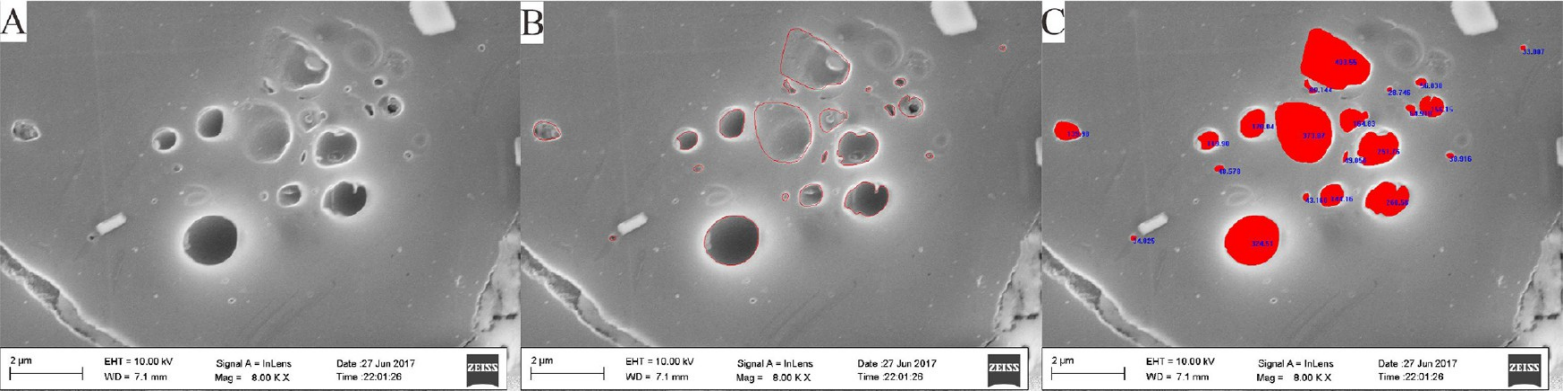
Process of image fractal. (A) the original SEM image. (B) The selected pores. (C) The obtained pore information.

The image fractals were determined using the Mandelbrot method.^24,49^ The FESEM images that were extracted using the Image J software provided the perimeter and area of pores that are required for the Mandelbrot method. The pores and minerals were divided into black and gray in the FESEM image. It was difficult to select accurate pores by adjusting the threshold of the gray value due to the uneven color of these images. We therefore manually delineated the pores to obtain more accurate pore information (Figure 4).

The Mandelbrot theory can be expressed as eq 6.
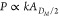
6

In eq 6, *P* is the pore perimeter, *A* is the pore area, *k* is a constant, and the fractal dimension *D_M_* can be obtained from the slope of log*P* and log*A*:
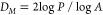
7

## 3 Results

### 3.1 Pore Classification

The current mainstream pore classification is based on the International Union of Pure and Applied Chemistry (IUPAC) standard. With this classification method, the pores are divided into three categories based on the IUPAC standard: macropores (>50 nm), mesopores (2–50 nm), and micropores (<2 nm).^50,51^

The different test methods can only characterize a partial range of pore sizes accurately due to experimental limitations. The MICP, LP-N_2_, and CO_2_ adsorption were therefore all used to test all the pores. The ranges of these three experimental tests differ but overlap and can therefore be used to study the full range of pore information. MICP can theoretically measure the pores of 5–177,923 nm.^40^ However, the identification of these pores is uncertain at low pressures. The minimum pore size that mercury can enter depends on the mercury injection pressure in the experiment. If the mercury injection pressures are too high, it will destroy the pore structure and cause errors in the results. Many studies have shown that the suitable pore size range for mercury intrusion experiments is greater than 50 nm.^52,53^ The BJH uses capillary condensation to analyze pores that range in size from 0.85 to 150 nm. This model of a low-pressure nitrogen adsorption experiment is based on the Kelvin equation. The calculation accuracy decreases for pores smaller than 2 nm and larger than 50 nm. To analyze pores ranging in size from 2 to 50 nm, nitrogen adsorption was mainly used, while data outside this range can be used as relative data for standard pore structures.^26,46,54^ CO_2_ can enter smaller pores than N_2_ because of its molecular properties. CO_2_ can therefore be used to analyze pore sizes less than 2 nm.^42,43^

**Figure 5:**
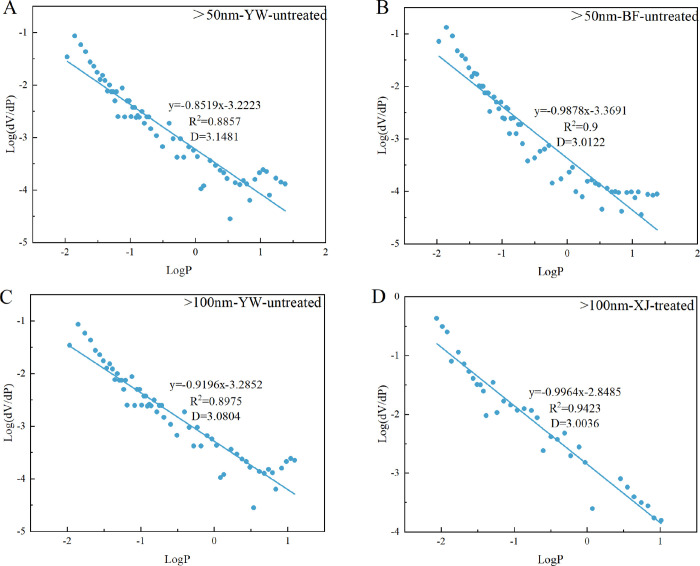
Pore range of samples with fractal dimensions over 3. (A, B) Fractal dimensions of the pores over 50 nm of the untreated YW and BF samples. (C) Fractal dimension of pores over 100 nm of the untreated YW sample. (D) Fractal dimension of pores over 100 nm of the treated XJ sample.

The fractal dimension is the most important parameter in fractal geometry theory and application. This index measures the complexity and irregularity of objects or fractals and can effectively characterize the irregularity and surface roughness of pores in porous media, including coal and shale.^41,53,55^ CO_2_ is mainly stored in coal seams via adsorption to the various pore surfaces. The pore morphology and surface characteristics are the main factors affecting CO_2_ adsorption. Understanding the effect of CO_2_ injection on fractal dimensions is therefore of great significance for CO_2_ storage. In this study, the pore size data were combined to calculate the fractal dimensions of the pores in different ranges. Most of the fractal dimensions exceeded 3 during calculations, irrespective of whether it was a pore larger than 50 nm or larger than 100 nm. The pore sizes are, however, of no significance to the fractals (Figure 5). Pores larger than 150 nm showed better fractal dimensions before and after the reaction, with the correlation coefficients being greater than 0.9 in all the cases. The detailed analysis can be found in Section 3.3.1 and Table 5. Since the pore structure characteristics match the fractal characteristic for the pores of the same pore size range, pore analyses were not discussed according to the traditional classification of macropores, mesopores, and micropores in this paper. The MICP data were used to analyze the changes in pore structure parameters and their fractal characteristics for pores over 150 nm. The pore structure characteristics of the 2–150 nm size were analyzed by LP-N_2_ adsorption data. Fractal dimensions that range from 2 to 3 were of importance (see Section 3.3.2 and Table 6). Pores smaller than 2 nm were characterized via CO_2_ adsorption data. The fractal dimensions of pores in this range are also significant (see Section 3.3.3 and Table 7).

**Table 5:** Fractal Dimensions of LP-N_2_ Adsorption, where *D*_3_ Represents 2–150 nm Pore Fractal Dimension and *D*_4_ Represents <2 nm Pore Fractal Dimension

sample	*A*_1_	*D*_3_	*R*^2^	*A*_2_	*D*_4_	*R*^2^
YW-untreated	–0.5044	2.4956	0.9761	–0.3229	2.6771	0.9176
YW-treated	–0.4543	2.5457	0.9987	–0.2323	2.7677	0.951
XJ-untreated	–0.5382	2.4618	0.9863	–0.5965	2.4035	0.8786
XJ-treated	–0.4234	2.5766	0.9908	–0.1984	2.8016	0.8191
BF-untreated	–0.5783	2.4217	0.9996	–0.6057	2.3943	0.9301
BF-treated	–0.4314	2.5686	0.9752	–0.1237	2.8763	0.7366

**Table 6:** Fractal Dimensions from CO_2_ Adsorption

	YW			XJ			BF		
sample	*K*	*D*_5_	*R*^2^	*K*	*D*_5_	*R*^2^	*K*	*D*_5_	*R*^2^
untreated	0.9117	2.7351	0.9995	0.8871	2.6613	0.995	0.9124	2.7372	0.995
treated	0.8837	2.6511	0.9953	0.9303	2.7909	0.997	0.8735	2.6205	0.9967

**Table 7:** Image Fractal Dimensions of the Pores before and after the Reaction

	calcite		kaolinite		pores in coal matrix	
sample	untreated	treated	untreated	treated	untreated	treated
YW	1.1624	1.0864	1.1306	1.1362	1.2336	1.2668
XJ	1.3536	1.0028	1.1282	1.0844		
BF	1.6602	1.2076	1.191	1.186	1.03	1.005

### 3.2 Changes in the Pore Structure

#### 3.2.1 >150 nm Pore Size

**Figure 6:**
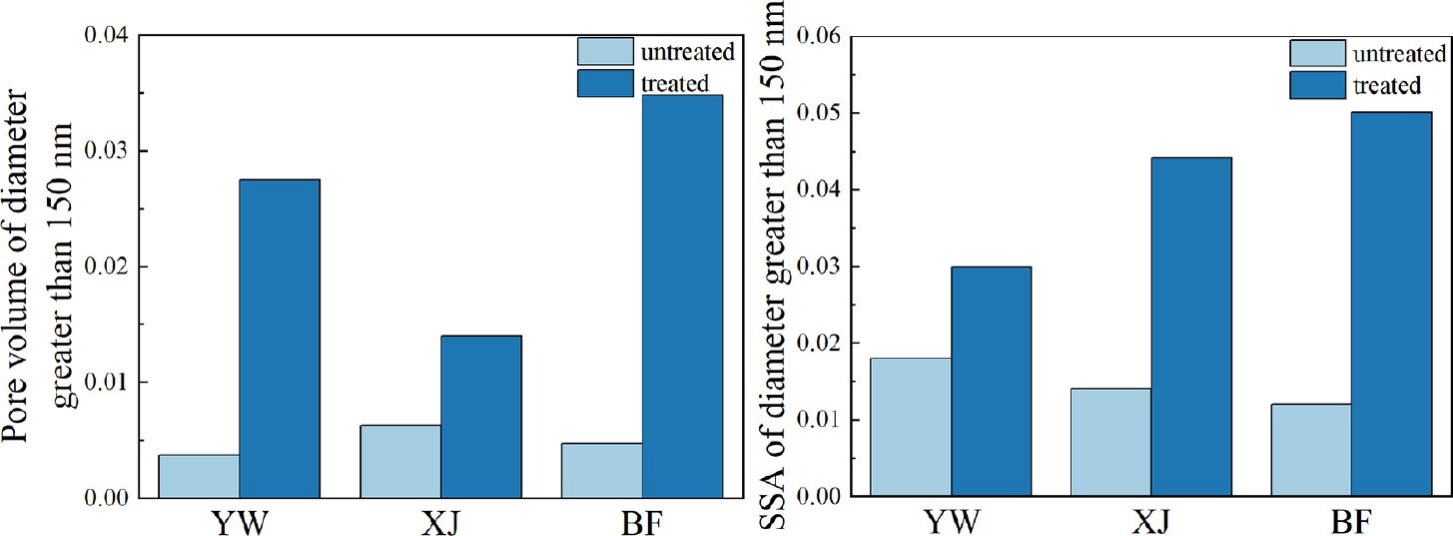
Changes in the pore volume and specific surface area of the pores larger than 150 nm before and after the reaction.

After the reaction, the pore volume and specific surface area of the pores larger than 150 nm in the three samples increased significantly. The degree of increase varied between the samples. The pore volume of the BF sample increased the most by 0.02373 mL/g, followed by the XJ sample, which increased by 0.00765 mL/g, and the YW sample, which increased the least by 0.03008 mL/g (Figure 6). The BF sample experienced the largest change in specific surface area and increased by 0.03807 m^2^/g, followed by the XJ sample, which decreased by 0.03021 m^2^/g, and the YW sample, which decreased the least by 0.01186 m^2^/g (Figure 6).

**Figure 7:**
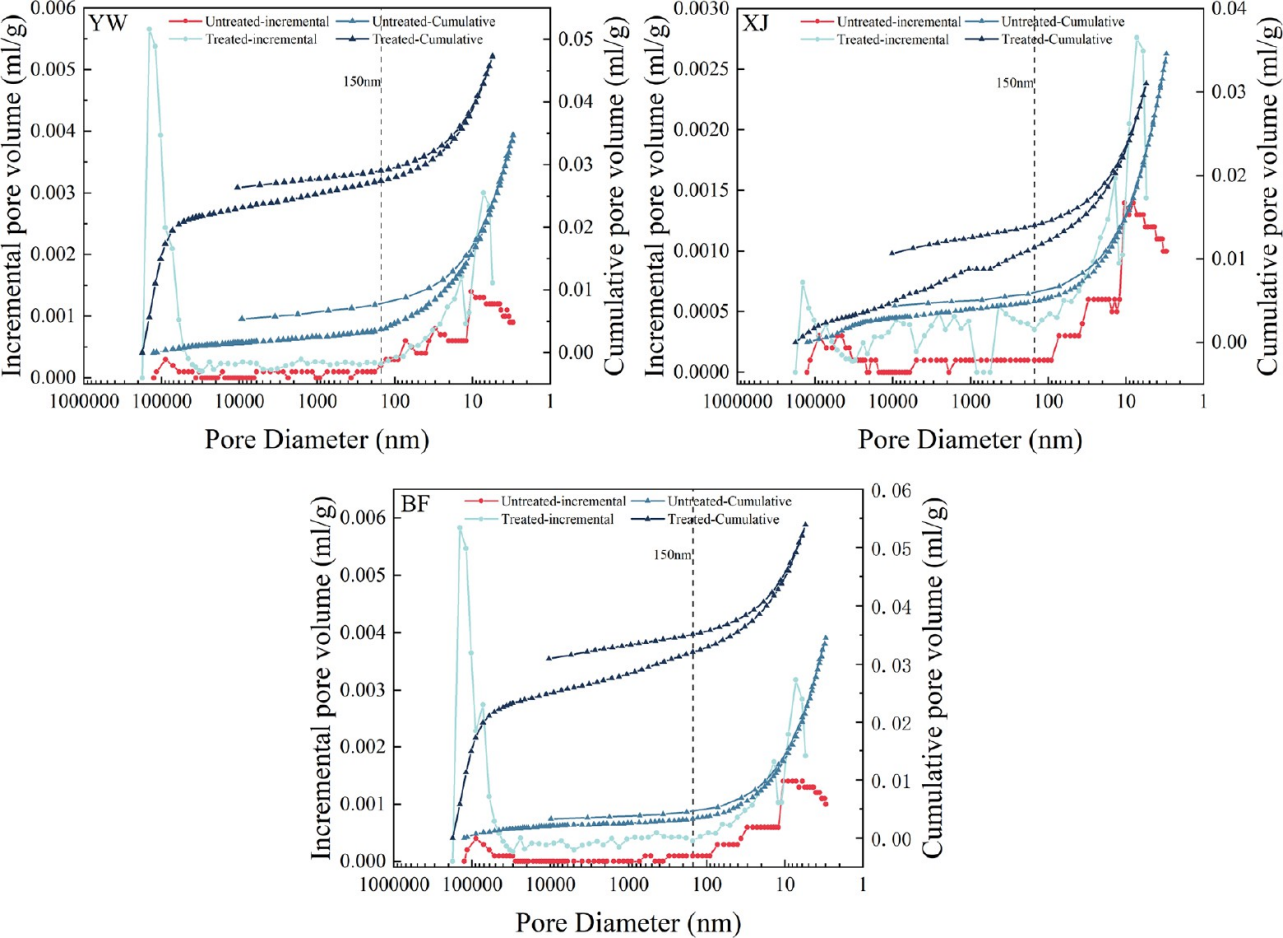
Pore distribution and mercury intrusion and extrusion curves before and after the reaction.

Figure 7 shows the mercury intrusion and extrusion curves of the three samples before and after the reaction. All the samples have mercury intrusion and extrusion curves that do not overlap and form a hysteresis loop. The hysteresis loop of coal samples can to a certain extent express the connectivity of pores. The better the connectivity of the sample, the closer the mercury intrusion and extrusion volumes are, and the narrower the hysteresis loop will be. The lower the connectivity, the greater the difference between the mercury intrusion and extrusion volumes, and the larger the hysteresis loop.

The pores in the coal samples can, therefore, be defined as effectively connected pores and noneffective connected pores. The changes in the content of effectively connected pores and noneffective connected pores before and after the reaction can therefore be analyzed qualitatively.^56^
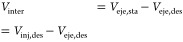
8
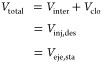
9
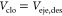
10

In eqs 8–10, *V*_inter_ is the effectively connected pore volume in mL/g, *V*_eje, sat_ is starting pore volume of mercury extrusion in mL/g, *V*_eje, des_ is the end pore volume of mercury extrusion in mL/g, *V*_inj, des_ is the end pore volume of mercury intrusion in mL/g, *V*_total_ is the total pore volume in mL/g, and *V*_clo_ is the noneffective connected pore volume in mL/g.

Except for the XJ sample, the pore connectivity increased in all the samples. After the reaction, meanwhile, the noneffectively connected pores also increased. The percentage of effectively connected pores decreased for the YW and BF samples, except for the XJ sample, which increased. It can therefore be inferred that, after the reaction, the effectively connected pore space of the coal samples will increase due to the increase in the overall pore volume. More noneffectively connected pores will be generated because of the decreases in their proportion. This poorer connectivity may be caused by the dissolution and precipitation of minerals. The more developed internal fractures in the XJ sample caused its inconsistent variation, which affects its fractal dimension to some extent.

After the reaction, the pore size distribution of all three samples changed significantly. All the treated samples increased in pore volume, and all were characterized by a bipolar distribution, with a significant increase in the pore content larger than 60 μm and smaller than 30 nm (Figure 7). These changes are particularly evident in the YW and BF samples. However, in the XJ sample, the number of pores larger than 60 μm was significantly smaller than in the YW and BF samples. The effect of ScCO_2_ on high-ranking coals in previous studies mainly concentrated in the micropores, as evidenced by the large increase in pore volume in the range of less than 30 nm. When ScCO_2_ is injected into coal, a reaction occurs between the water and rock, which dissolves and precipitates minerals, especially calcite. These rock–water interactions are the main reason for the increase in pore space.^35,36^ It can be inferred that, after ScCO_2_ injection, the original unopened pores will be transformed into open pores due to the dissolution and precipitation of minerals. This, combined with the changes in the connected pores, will cause an increase in effectively connected pores. At the same time, more noneffectively connected pores will be generated, and the increase is much larger than the effectively connected pores.

#### 3.2.2 2–150 nm Pore Size

**Figure 8:**
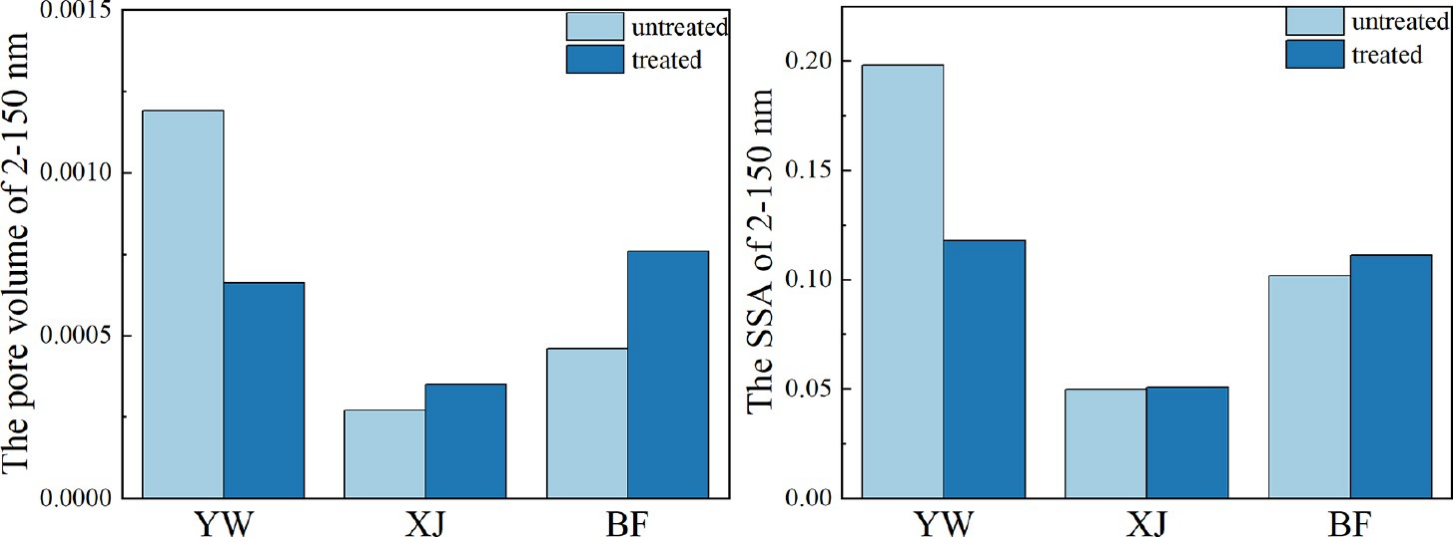
Changes in the pore volume and specific surface area of the pores within the 2–150 nm range before and after the reaction.

**Figure 9:**
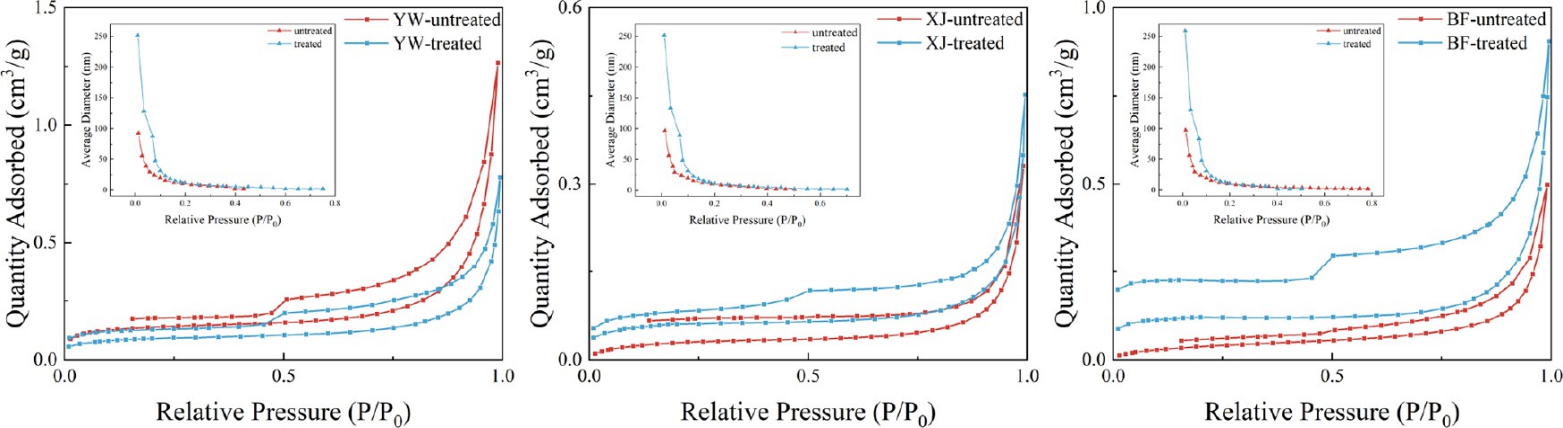
LP-N_2_ adsorption–desorption curves and pore radius changes before and after the reaction.

The pore volume of the XJ and BF samples increased after the reaction in the pore size range of 2–150 nm. The pore volume of the YW sample however decreased. The changes in specific surface area and pore volume of the three samples were consistent (Figure 8). The adsorption volumes increased for both the XJ and BF samples, but not the YW sample. Figure 9 shows the adsorption and desorption curves of the three samples. The adsorption volume of sample YW decreased after the reaction, which corresponds to the change in their pore volumes. The adsorption and desorption curves did not overlap for any of the three samples. The adsorption and desorption curves and morphology of the hysteresis loop provide more information on the pore morphology. The adsorption curve has some adsorption capacity at low pressures. This curve becomes steeper when the relative pressure is close to 1.0 and the adsorption saturation is not reached. The desorption curve is also steeper at high pressures, and the hysteresis loop formed by the adsorption curve and desorption curve is relatively narrow. The three samples in our study belong to the H3 curves and have H4 characteristics based on the IUPAC classification of adsorption and desorption curves.^54,57^ The pore structure contains mainly slit-shaped pores, wedge-shaped semiclosed pores, and a small amount of ink bottle-shaped pores.

**Figure 10:**
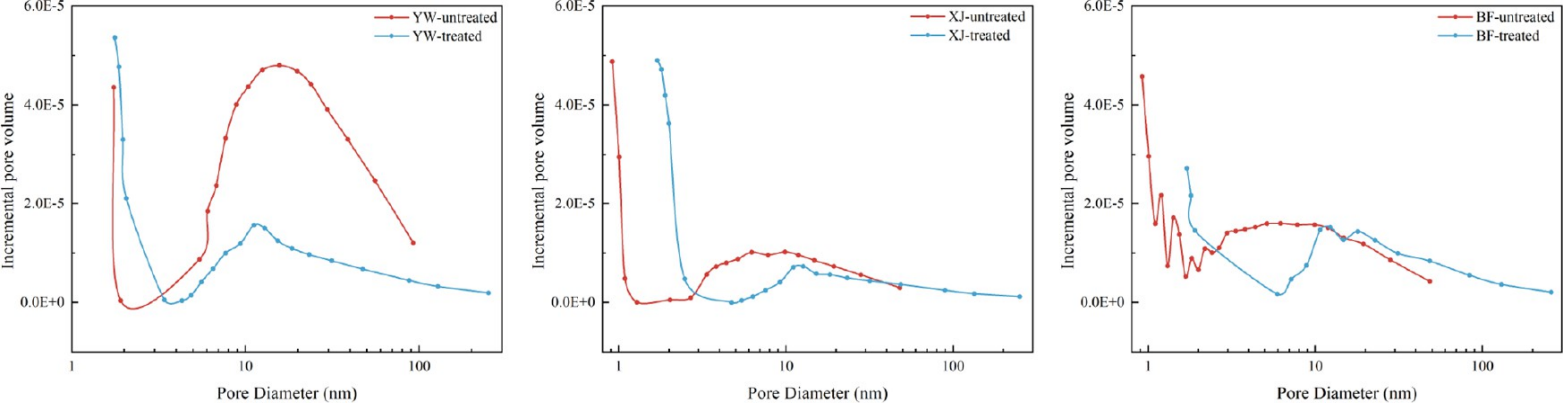
Pore distribution from LP-N_2_ adsorption before and after the reaction.

After the reaction, the pore size distribution of the three samples changed significantly. Pores within the 2 nm pore size decreased but increased in the range greater than 2 nm for all the samples, except for the YW sample. The pores of the YW sample that were greater than 3 nm were smaller after the reaction than in the original sample (Figure 10), which is consistent with the change in its pore volume. The pore distribution changes indicate that new pores are generated during the reaction compared to the original pores. The reduction of pores in the YW sample after the reaction may be the result of the increase of the original pores into a larger pore size range, as well as the blockage of pores caused by the precipitation of minerals.

#### 3.2.3 <2 nm Pore Size

**Figure 11:**
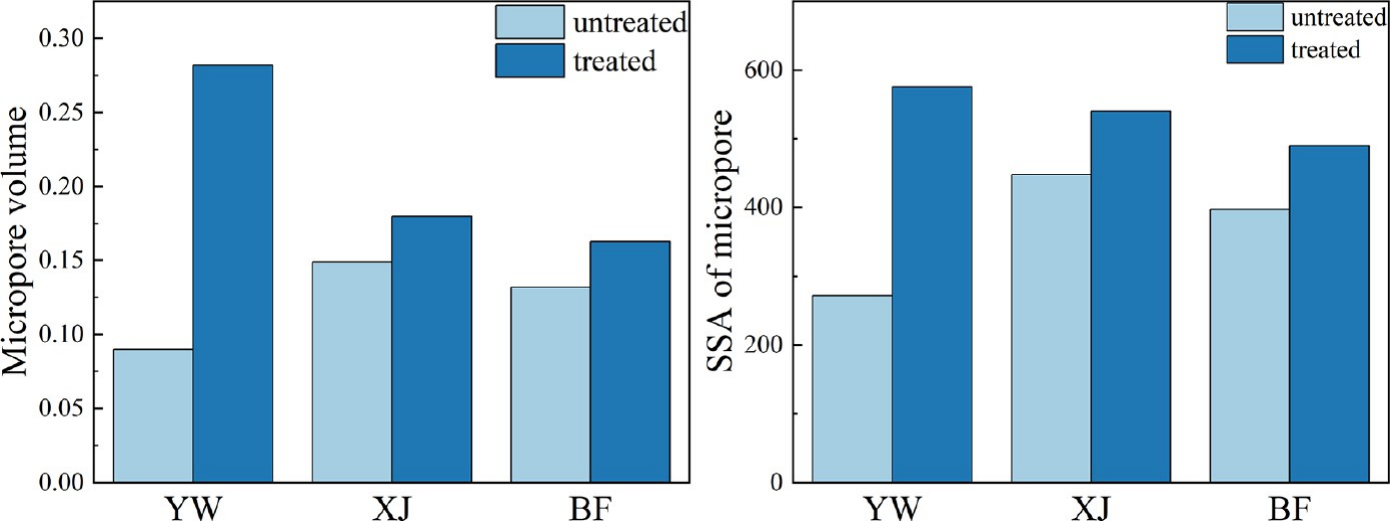
Changes in pore volume and specific surface area of >2 nm pores before and after the reaction.

The pore volume of the micropores increased after the reaction, especially for the YW sample. Here, the pore volume increased by 0.192 mL/g compared with the original, while for the XJ and BF samples, the pore volume increased by 0.031 mL/g (Figure 11). The change in the specific surface area is the same as the change in pore volume. The specific surface area of the YW sample increased the most at 575.563 m^2^/g, while the specific surface area of the XJ and BF samples increased by 91.677 and 92.925 m^2^/g, respectively (Figure 11). The changes in the specific surface area are comparable to the change in pore volume. The change in the micropores was the main driver for changes in the specific surface area. Changes in the specific surface area of macropores and mesopores are negligible compared to the micropores. The variation in specific surface area and pore volume of pores in this range explains the variation in total specific surface area and volume, which can mainly be attributed to changes in the pore volume, with more pores causing an increase in the specific surface area.

**Figure 12:**
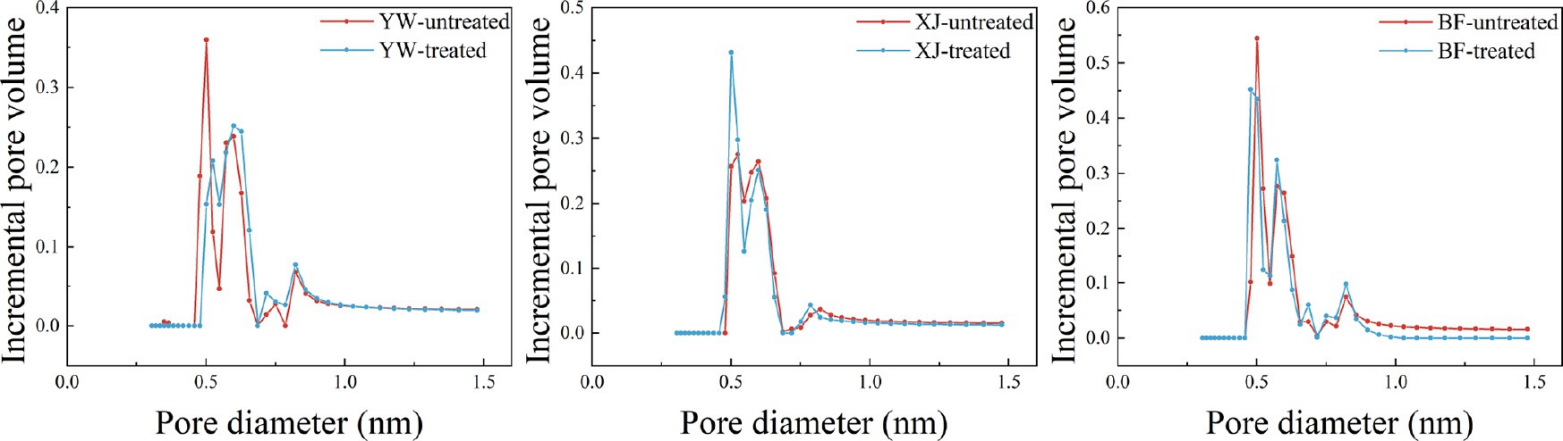
Pore distribution from CO_2_ adsorption before and after the reaction.

The pore size distribution also showed obvious changes after the reaction. Three peaks could be observed in the pore size distribution of the samples before and after the reaction, at 0.5, 0.6, and 0.8 nm. No obvious rule can however explain these changes. The pore sizes of the YW and BF samples both decreased at 0.5 nm and increased at 0.6 and 0.8 nm. At 0.5 nm, the pore sizes of the XJ sample increased significantly, while it changed little at 0.6 and 0.8 nm (Figure 12). These results indicate that the size increase in the micropores is not only caused by the additional new generation of pores but also by the size increase of the original pores.

### 3.3 Changes in Fractal Dimensions

#### 3.3.1 >150 nm Pore Sizes

**Figure 13:**
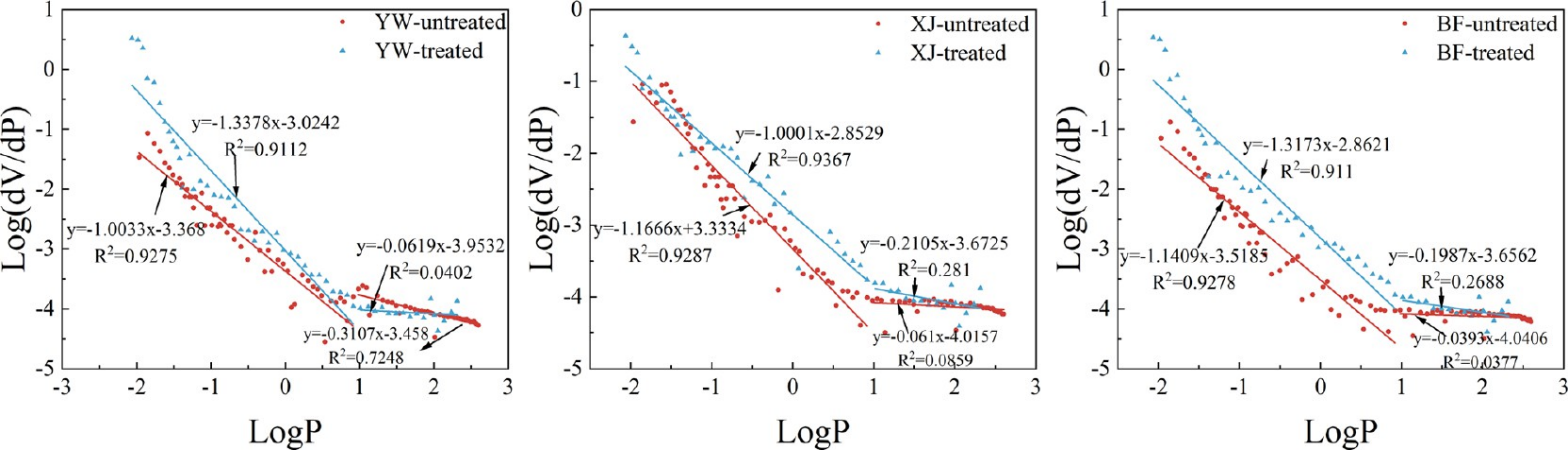
Fractal dimensions of MICP.

The pressure corresponding to the 150 nm pore size is about 8.25 MPa in the MICP. The fractal dimensions are calculated based on this pressure point as the boundary. In this study, *D*_1_ represents the fractal dimension of pores >150 nm, and *D*_2_ is the fractal dimension of pores <150 nm. The fractal dimensions before and after the reaction in this range are greater than 3, and the correlation coefficients are all less than 0.9, which does not have fractal significance (see Figure 13 and Table 4). The *D*_1_ ranged between 2 and 3 before and after the reaction in the range of >150 nm, and all the results correlate at more than 0.9. These results prove that the fractal results are meaningful and can be used as reliable evidence for pore analysis. The fractal dimensions of the YW and BF samples both decreased after the reaction, by 0.3345 and 0.1764, respectively, while the fractal dimension of the XJ sample increased by 0.1655 after the reaction.

The fractal dimensions of the pores larger than 150 nm decreased after the reaction. Previous studies showed an increase in the fractal dimensions of coal pores after the reaction.^31,58^ The fractal dimensions of all the samples in this experiment decreased after the reaction, except the XJ sample. Once the ScCO_2_ is injected, it dissolves in water and subsequently reacts with the coal minerals. The subsequent dissolution and precipitation of minerals are the main reason for the changes in the pore structure and the changes in the fractal dimensions. A portion of the calcite will react with it and undergo complete dissolution after ScCO_2_ injection. The surrounding coal matrix will however remain unaffected. This causes the preservation of the original calcite particle morphology within the newly created pores. The fractal dimensions of the YW and BF samples reduce because the morphologies of these newly generated pores are mostly controlled by the calcite particles and because the newly generated pores have similar morphologies and are more uniform. The increase in the fractal dimension of the XJ sample may be caused by their more developed endogenous fractures. There were still more effectively connected pores than noneffectively connected pores after the reaction. The more effectively connected pores constitute a more complex pore system, which increases the fractal dimension.

#### 3.3.2 2–150 nm Pore Sizes

**Figure 14:**
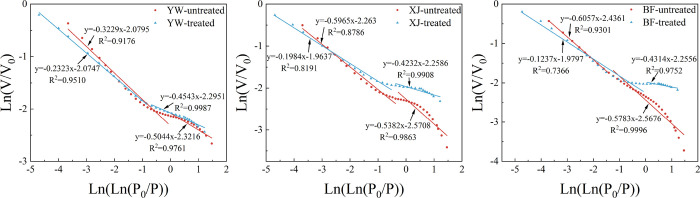
Fractal dimensions of LP-N_2_ adsorption.

The fractal dimensions of the nitrogen adsorption data were calculated using the 2 nm pores as the cutoff point. The relative pressure corresponding to the 2 nm pores for the different samples differed slightly, but the relative pressure mostly remained around 0.5–0.7. The pressure data that correspond to the different sample pore size data were used to calculate the fractal dimensions separately (see Figure 14 and Table 5). *D*_3_ represents the fractal dimension of 2–150 nm pores, with good correlation coefficients greater than 0.9 and with good fractal characteristics. *D*_4_ represents the fractal dimension of pores <2 nm, which all range between 2 and 3, and where some of the correlation coefficients are below 0.9.

The fractal dimensions of pores within the 2–150 nm size increased after the reaction and are related to the maturity of the samples. The YW sample increased by 0.0501, while the XJ sample increased by 0.1148, and the BF sample increased by 0.1469. The maturity of these three samples ranges from low to high. As the maturity increases, the fractal dimensions also increase, together with an increase in the homogeneity and roughness of the pores. The surfaces of mesopore and macropores also however become rougher. A variety of pores with different morphologies are developed during the incomplete dissolution of calcite produces. This is one of the reasons for the increase in the fractal dimensions. The fractal dimensions similarly increase in pores with sizes less than 2 nm. The results of the micropore data measured by nitrogen adsorption are not reliable as the sample only has some large pores. This data can therefore only be used as a reference for the characteristics of micropore fractals.

The fractal dimensions of the pores with sizes of 2–150 nm differ significantly from the fractal dimensions of pores larger than 150 nm. The pore volume and specific surface area of the XJ and BF samples both increased significantly after the reaction due to the combined changes in the pore volume and specific surface area. There was also an increase in the maturity of the samples. The fractal dimension of the YW sample increased even though the pore volume and specific surface area decreased after the reaction. The changes that were observed can be ascribed to the increase in pore size after the reaction of the existing pores. The precipitation of various minerals also blocked some pores. The number of enlarged pores in the YW sample is significantly greater than the number of newly formed pores even though these changes occur in all the samples. This caused a decrease in the pore volume and specific surface area of the YW sample. The increase in the fractal dimensions indicates an increase in the complexity of the original pores after the reaction. However, the new pores formed by the complete dissolution of the minerals are very uniform, which would reduce the fractal dimensions. This premise was subsequently supported by the fractal results of FESEM images.

#### 3.3.3 <2 nm Pore Sizes

**Figure 15:**
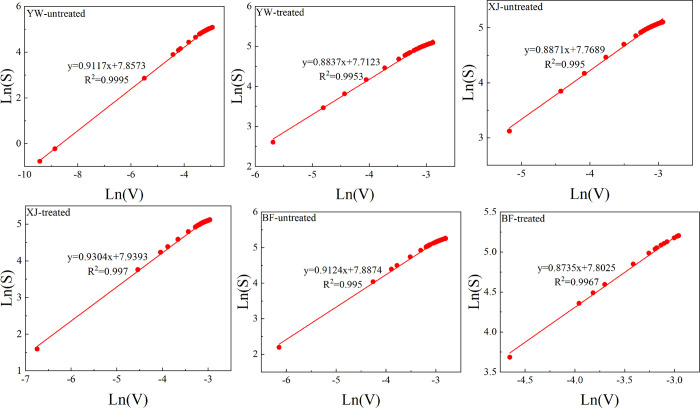
Fractal dimensions of CO_2_ adsorption.

Figure 15 and Table 6 show the micropore fractal results. The fractal dimensions of the YW and BF samples decreased by 0.084 and 0.1167, respectively, while the fractal dimension of the XJ sample increased by 0.1296 after the reaction. The pore volume and specific surface area of all three samples increased after the reaction. ScCO_2_ had the greatest impact on the micropores of high-ranking coal. Here, the injected ScCO_2_ formed new pores and enlarged the original pores. The pore volume of the YW sample decreased in the range of 2–150 nm. The increase in the pore volume was the largest in the range of less than 2 nm. This indicates that the original pores in this sample are affected by the degree of reaction. A smaller increase in pore size caused a smaller number of pores to move from the small pore size range to the larger pore size range. Therefore, the range of micropores after the reaction includes the original pores that increased but that did not enter the larger pore size range and newly generated micropores. The fractal dimension of the newly generated pores may be small, while the fractal dimension of the original pores may increase after the reaction. The fractal dimension of the YW sample decreased less than the BF sample. This is because some of the original pores did not increase too much, causing the fractal dimensions of these pores to be larger than that of the newly generated pores. The YW sample, therefore, shows a small change in the fractal dimension in the micropore range. The increase in the fractal dimension of the XJ sample may also be related to the properties of the samples. The more brittle nature of the XJ sample causes it to generate more micro fractures after the reaction, resulting in a more complex pore system, which increases the fractal dimension. The fractal dimensions increased after treatment in previous studies on the changes in fractal dimensions before and after CO_2_ treatment. However, in our study, the fractal dimension did not cause an increase in all of the fractal dimensions. The reasons for this were the different maturities of the samples and the different experimental conditions. Liu et al. selected coals of different grades, and the fractal dimension was not calculated according to the correspondence of the pore size data.^31^ Wen et al. carried out the experimental conditions of liquid CO_2_ treatment at −40 °C, and CO_2_ was not in a supercritical state.^58^

### 3.4 Variation in Image Fractals

**Figure 16:**
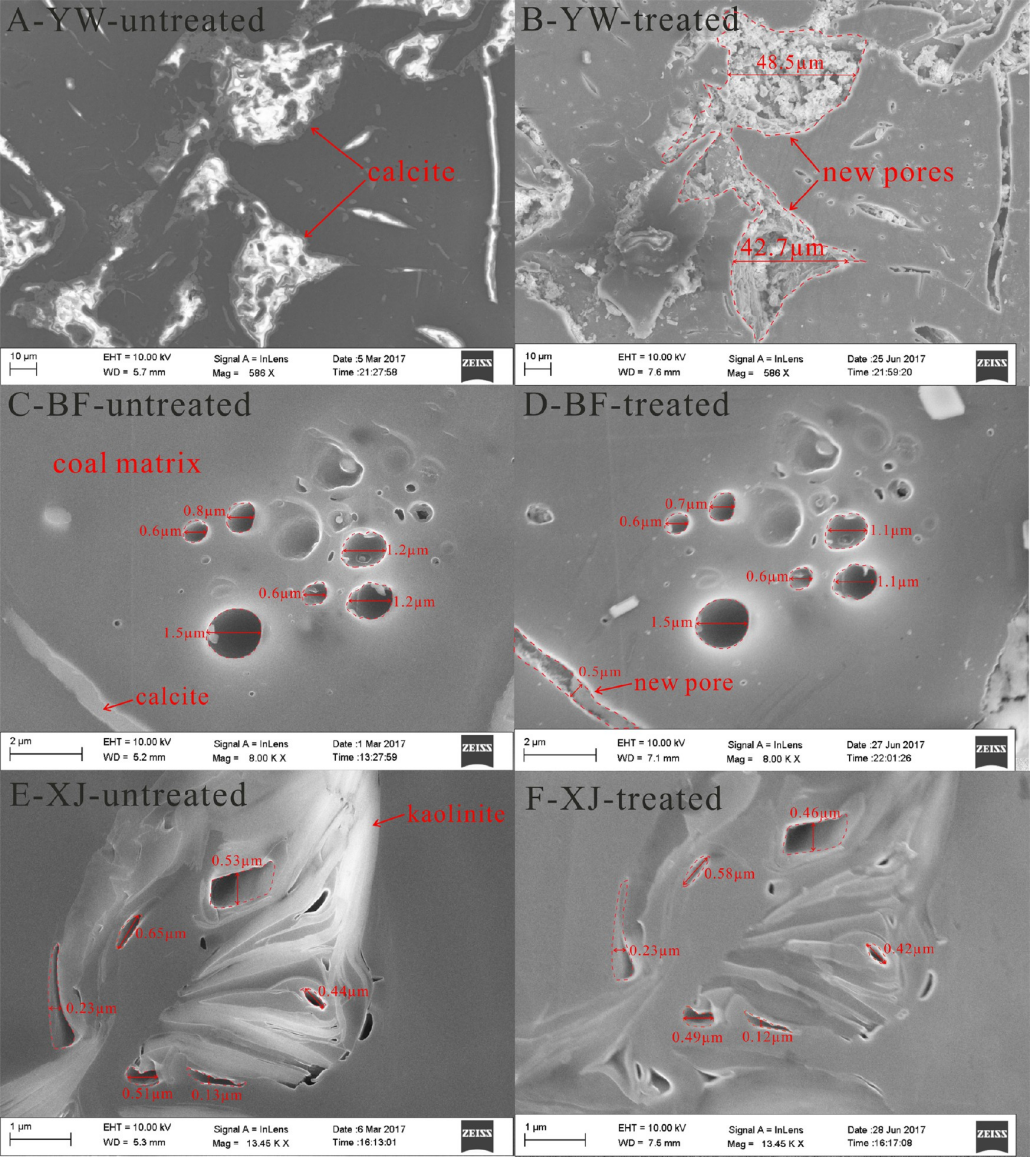
FESEM images of the pore characteristics in calcite (A, B), coal matrix (C, D), and kaolinite (E, F) before and after the reaction.

Coal pores include various mineral pores and coal matrix pores. FESEM images can be sued to determine the area and perimeter information of different pore types. This allows for a study of the effect of ScCO_2_ injection on different pores based on the variation in its fractal dimensions. Statistical analyses show that the pores in the coal after the reaction are mainly formed by calcite dissolution (Figure 16A,B), followed by pores in the coal matrix (Figure 16D), and by pores in the different clay minerals (Figure 16E,F). Since SEM images are two-dimensional, only the mineral surface can be observed. Therefore, the complete dissolution of the mineral was determined by the observable mineral surface. After the reaction, the mineral surface cannot be observed in the same position that is complete dissolution but also can be observed as incomplete dissolution.

**Figure 17:**
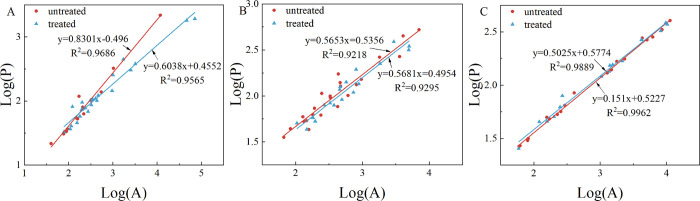
Calculations of the image fractal. (A) The fractal dimensions of the pores before and after calcite dissolution for the FESEM same view. (B) The fractal dimensions of the pores before and after the reaction in the coal matrix. (C) The fractal dimensions of the pores before and after the reaction in kaolinite.

The largest changes in the fractal results observed after the reaction were in the pores that formed when the calcite particles were dissolved. The pores of the coal matrix and various clay minerals did not differ significantly before and after the reaction. Most calcite particles were dissolved after the reaction. This dissolution of particles was the major reason for the increase in pore volume. In addition, a large quantity of dissolved calcite, which connected the previously disconnected pores, caused the increase in pore connectivity of the samples. The selected FESEM images in each sample were taken in the same area before and after the reaction. The area and perimeter of the pores in the visual field of the FESEM image before the reaction and the area and perimeter of the pores in the same visual field after the reaction were counted. The fractal dimensions of the pores before and after the reaction were from this information calculated. A total of 484 data points on different types of pores were counted (see Figure 17 and Table 7).

The largest changes in fractal dimensions were observed in the pores that formed after the dissolution of calcite. All the pore types showed small changes in the fractal dimension before and after the reaction, including pores in coal, and pores in and near kaolinite. The fractal dimensions of the pores formed by dissolving calcite in the same field of view of the three samples have all declined. Levels of decline gradually increase with increasing coal maturity. The fractal dimensions from low to high maturity decreased by 0.076, 0.3508, and 0.4526, respectively. The fractal dimensions of pores in the kaolinite and the coal matrix differ a little before and after the reaction. Only the XJ sample changed only slightly after the reaction in the kaolinite and its edge pores, where the fractal dimension was reduced by 0.0438. Compared with the changes caused by calcite dissolution, these changes can be ignored. The changes in the other two samples are all around 0.005, and here too, the overall change can be ignored. The change of pores in the coal matrix is between 0.02 and 0.03, which can also be ignored.

Most of the FESEM images cover large viewing areas, and the statistics of fractal dimension mainly represent the fractal dimensions of large-aperture pores. The fractal dimensions that were calculated from the MICP in the large aperture range also decreased for all the samples, except for the XJ sample. This is due to the increase in the number of effectively connected pores in the XJ sample after the reaction, which ultimately increased the complexity of the pore structure.

## 4 Discussion

### 4.1 Effect of Mineral Changes

**Figure 18:**
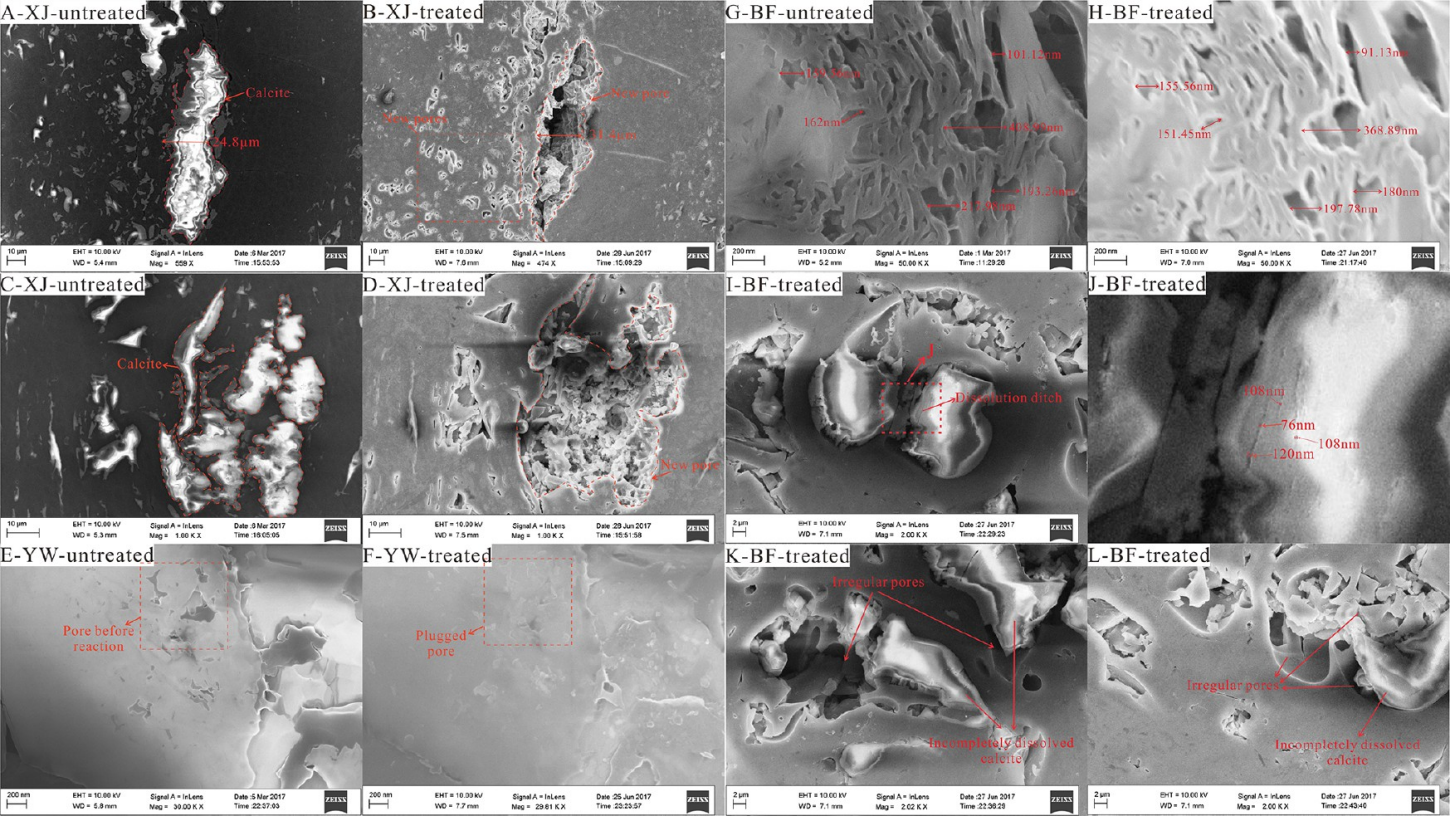
Effect of mineral changes on the pore structure due to FE-SEM. (A, B) Massive dissolution of calcite. (C, D) Pore connectivity is caused by calcite dissolution. (E, F) Part of the pores in kaolinite is blocked after the reaction. (G, H) Blocked pores and pores with reduced diameter after the reaction. (I, J) Dissolution ditches formed by incomplete dissolution of calcite. (K, L) Irregular pores formed by incomplete dissolution of calcite.

In the previous study, the dissolution of large particles of calcite was the main reason for the increase in the full-size pore volume.^36,56^ In the image with higher resolution, a large amount of calcite that dissolved after the reaction is visible. A large number of macropores and mesopores also formed after the dissolution, which caused an increase in the pore volume (Figure 18B). The complete dissolution of isolated calcite particles can increase the connectivity of the macropores and mesopores. In addition, some pores are hidden due to carbonate that fills the pores before the reaction. The intercrystalline pores of organic matter that are exposed after dissolution will also increase the partial connectivity of the pores (Figure 18C,D), thereby increasing the volume of effectively connected pores. In addition to all the dissolved calcite, there are still many incompletely dissolved calcites in the coal. After the dissolution of these calcite particles, many dissolution ditches formed on the surface, which changed the pores in the range of 2–150 nm (Figure 18I,J). The injection of ScCO_2_ will not only dissolve large amounts of calcite, but a series of reactions will also occur. This series of reactions are often accompanied by the dissolution of the original minerals and the generation of new minerals. These reactions, therefore, not only increase the pore volume but also reduce the volume of some pores and may completely block some pores (Figure 18E–H). Based on the change in the overall pore volume after the reaction, the increase in the pore volume is much greater than the pore blockage.

The image fractal dimensions of the pores that formed after the complete dissolution of the calcite surface decreased. The MICP fractal dimensions of YW and BF also decreased after the reaction, which was consistent with the image fractal dimensions. This indicates that the reduction of the fractal dimensions of the pores generated after the complete dissolution of the calcite surface is the main reason for the reduction of the fractal dimensions in the MICP. This reduction in the fractal dimension indicates that the surface of the newly generated pores has a lower roughness and complexity than the original pores due to calcite dissolution. Calcite in coal is derived from the plants that are present during its formation, and its mineral morphology is influenced by the cellular state of the original plant. As a result, the pores that formed after complete dissolution exhibit comparable morphology. Furthermore, the incompletely dissolved calcite will form pores with different morphologies (Figure 18K,L). The space left after its dissolution is completely random and not controlled by any factor, which increases its complexity. The fractal dimensions of the pores in the range of 2–150 nm, therefore, increased. The FESEM images show no obvious change for other types of minerals and pores. The changes in the fractal dimensions are also small, which means its effect on the fractal dimensions of the overall pores can be ignored.

### 4.2 Pore Changes at Different Stages

The Pore volume, specific surface area, and fractal dimensions for the different pore ranges changed significantly after the reaction (Table 8). For changes in macropores larger than 150 nm, the pore volume increased by 641.41% for the YW sample, 121.38% for the XJ sample, and 639.98% for the BF sample. For the YW sample, the specific surface area increased by 65.91%; for the XJ sample, it increased by 215.76%, and for the BF sample, it increased by 317.25%. The fractal dimension decreased by 11.16% for the YW sample and by 4.86% for the BF sample. It however increased by 5.88% for the XJ sample. The pore characteristics of the YW and BF samples followed similar patterns, but the YW sample had the lowest increase in specific surface area and a greater decrease in fractal dimension. The FESEM shows that the complete dissolution of the calcite surface results in the creation of many pores larger than 150 nm. The morphologies of the newly generated pores are controlled by the original cell morphologies of the calcite filling. As a result, the pore shapes are more similar, the pore edges are smoother, and the image fractal dimensions are reduced after the reaction (Figure 17A,B). As the YW sample has a higher calcite content than the BF sample, its fractal dimension is reduced even more. The XJ sample has the lowest elevation in pore volume, but a higher elevation in specific surface area. The calcite content of the XJ sample is similar to that of the BF sample. The BF sample also has a large number of pores formed by complete calcite dissolution (Figure 18A–D), but the overall fractal dimension is still elevated. However, the MICP results show that the XJ sample has the largest increase in its effectively connected pore volume (Table 3). The pores produced by calcite dissolution are more often noneffectively connected pores (Figure 18K,L). Therefore, the increase in the effectively connected pore volume is due to the generation of microfractures. As a result, the strong brittleness of the XJ sample caused the development of endogenous fractures, led to a more complex pore system, and increased its fracture dimension trends after the reaction.

**Table 8:** Percentage Change in Pore Volume, Specific Surface Area, and Fractal Dimension of Pores at Different Pore Ranges after the Reaction

pore size	>150 nm			2–150 nm			<2 nm		
sample	YW	XJ	BF	YW	XJ	BF	YW	XJ	BF
pore volume	641.41%↑	121.39%↑	639.98%↑	44.54%↓	29.63%↑	65.22%↑	213.33%↑	20.81%↑	23.48%↑
specific surface area	65.91%↑	215.76%↑	317.25%↑	40.54%↓	1.87%↑	9.21%↑	212.04%↑	20.46%↑	23.42%↑
fractal dimension	11.16%↓	5.88%↑	4.86%↓	2.01%↑	4.66%↑	6.07%↑	3.07%↓	4.87%↑	4.26%↓

The change in pore volume was inconsistent for pores between 2 and 150 nm. The YW sample decreased by 44.54%, the XJ sample increased by 29.63%, and the BF sample increased by 65.22%. The change in the specific surface area was consistent with the change in the pore volume, with the YW sample decreasing by 40.54%, the XJ sample increasing by 1.87%, and the BF sample increasing by 9.21%. The change in pore-specific surface area in this pore size stage is therefore mainly due to the change in pore volume. The reduction in the pore volume of the YW sample at this pore range may be caused by the expansion of pores. These pores belong to the 2–150 nm pore range before the reaction but belong to pores larger than 150 nm after the reaction. Also, these pores account for a large proportion. The fractal dimension increased by 2.01% for the YW sample, 4.66% for the XJ sample, and 6.07% for the BF sample. A variety of factors lead to the irregularity of pore structure changes in this pore size range. The increase in fractal dimension is related to the increase in the roughness of the mesopores and some macropores and the incomplete dissolution of calcite (Figure 18I,J) after the reaction.

The variation regular in pore structure characteristics of pores smaller than 2 nm was similar to that for pores larger than 150 nm. The pore volumes all increased after the reaction, with the YW sample increasing by 213.33%, the BF sample by 20.81%, and the XJ sample by 23.48%. The changes in the specific surface area were consistent with the pore volumes, and the increases were similar. The variation in the fractal dimension was variable, with the YW sample decreasing by 3.07%, the BF sample decreasing by 4.87%, and the XJ sample increasing by 4.26%. The reduction of the fractal dimensions proves that the newly generated micropores have similar morphologies. The increased fractal dimension of the XJ sample may still be related to the strong brittleness, resulting in a more sensitive response to ScCO_2_. A large number of newly formed micropores is conducive to the adsorption sequestration of CO_2_ in coal reservoirs.

### 4.3 Implications of Water

During the CO_2_-ECBM process in deep coal seams, the injected CO_2_ will compete with the CH_4_ in the coal seam for adsorption, thus improving the recovery rate of coalbed methane. However, CO_2_ injection will cause the adsorption expansion effect of the coal matrix, resulting in fracture closure and permeability attenuation.^59^ Therefore, the difficulty of CO_2_ injection in coal is mainly caused by the adsorption and expansion of the coal matrix.

The presence of water, on the other hand, may increase the potential for CO_2_ injection into coal reservoirs. Experimental studies have shown that the presence of water can lead to the creation of new pores and fractures and increase the permeability of high-ranking coals. As the injected CO_2_ combines with water, it produces carbonic acid, which dissolves the carbonate minerals in the coal.^60^ A similar phenomenon is present in low-grade coals. CO_2_-H_2_O systems increase the dissolution of minerals and the release of mineral surface ions in the low-grade coal samples and change the pore structure of coal more significantly than that of a single CO_2_ fluid.^61^ In addition, a single CO_2_ fluid also weakens the strength of the coal, while the coupling effect of the CO_2_-H_2_O causes greater intensity changes, which can lead to more fracture generation of underground stress.^62^

CO_2_ injection can therefore be considered as a permanent sequestration method, which can also enhance the recovery of CBM reservoirs that are at the end of their development life. The predevelopment of the target layer should preferably be hydraulically fractured so that more water will remain in the reservoir. Alternatively, alternate injections of CO_2_ and water can be considered to increase the amount of CO_2_ that can be injected into the coal seam.

## 5 Conclusions

In this study, three kinds of coals with different maturity grades were treated with ScCO_2_ under simulated formation temperature and pressure conditions. The study used MICP and gas adsorption experiments to calculate the boundary points with fractal significance. The fractal dimensions were obtained by combining the pore volume, the specific surface area, information on the mineral changes in the SEM images, and the perimeter and area of the extracted pores. Thereafter, the structure and fractal characteristics of the pores were analyzed for different ranges, and the following conclusions are drawn:

(1) The injection of ScCO_2_ will increase the pore volume and specific surface area. It cannot only create new pores but also reshape the original pores and even enlarge or block the original pores. Many new pores and fractures can form in coal that is subjected to ScCO_2_-H_2_O injection. Therefore, the presence of H_2_O may increase the potential for the injection of CO_2_ into the coal seam. The amount of CO_2_ that can be injected into the coal seam may be increased by the injection of CO_2_ for sequestration after hydraulic pressure, or by the alternate injection of CO_2_ and H_2_O.
(2) The ScCO_2_-H_2_O mainly causes the dissolution of calcite and simultaneously increases the pore volume. The main reason for the reduced fractal dimensions of the pores larger than 150 nm is the strong morphological similarity of the pores formed by the complete dissolution of calcite. The complex morphology of the pores formed by the incomplete dissolution of calcite can cause an increase in the fractal dimension of the pores in the range of 2–150 nm.
(3) The fractal dimensions of the pores larger than 150 nm and smaller than 2 nm are mainly decreasing because many newly formed pores have a strong similarity. However, the strong brittleness of the sample may also cause the formation of a more complex pore network after the reaction and eventually lead to the increase of the fractal dimensions. The fractal dimensions of the 2–150 nm pores increase under the combined influence of several factors, indicating that the pore morphologies are more complex and the pore surface is rougher after the reaction.
